# AI-Powered Documentation for Mental Health Providers: Retrospective Observational Mixed Methods Study

**DOI:** 10.2196/84628

**Published:** 2026-03-12

**Authors:** Kaitlin E McCrudden, Mackenzie S Swirbul, Emily E Peake, Michael J Rodio, Aarthi Padmanabhan

**Affiliations:** 1 Talkspace, 2578 Broadway #607, New York, NY, 10025, United States, 1 888-846-4821

**Keywords:** artificial intelligence, AI, generative AI, AI scribe, digital health, telehealth, natural language processing, mental health, documentation, productivity, provider burnout

## Abstract

**Background:**

Mental health providers (MHPs) face a significant administrative burden from documentation, which can contribute to burnout and reduce time available for direct patient care. Although artificial intelligence (AI)–powered scribes have shown promise in general medical settings, their utility has not been well explored in the specific context of mental health care. This study describes the development and preliminary observational evaluation of Smart Notes, a generative AI tool designed to assist MHPs with documentation on a commercial virtual mental health platform.

**Objective:**

This study aimed to examine MHPs’ use and uptake of a generative AI documentation tool, including use patterns, perceptions of note quality, feedback and satisfaction, and changes in productivity.

**Methods:**

Smart Notes was developed using a HIPAA (Health Insurance Portability and Accountability Act)-compliant Azure OpenAI infrastructure to securely generate session summaries from individual therapy sessions. The tool was rolled out to MHPs in a phased approach, and its use required MHP and client consent as well as a mandatory review and edit by the MHP. We conducted a 1-year retrospective observational evaluation of the feature, examining MHP use, MHP-rated note quality, MHP feedback and satisfaction, and MHP productivity (weekly working hours, session completion rate, and client caseload).

**Results:**

Over 1 year, 162 full-time and 1366 contractual MHPs used Smart Notes to generate over 286,000 clinical notes. Use of the feature was high and stable, with nearly all (averaging 94% each week, SD 1%) full-time MHPs and most (averaging 72% each week, SD 6%) contractual MHPs using it weekly for eligible individual therapy sessions after the full launch. MHP-rated note quality was overwhelmingly positive, with 97.7% (9980/10,219) of feedback ratings among full-time MHPs and 98.4% (19,977/20,300) of feedback ratings among contractual MHPs being a “thumbs-up.” Qualitative feedback from MHPs was also largely positive, praising the tool for saving time and easing administrative burden. Finally, both full-time and contractual MHPs demonstrated changes in productivity.

**Conclusions:**

Our findings suggest that AI-powered documentation tools, such as Smart Notes, are a feasible and acceptable approach to supporting MHPs. The high adoption rate and favorable MHP feedback indicate the potential utility of these tools without compromising note quality. This study provides preliminary data regarding the application of AI documentation in the mental health care context, highlighting a promising path for future research and development in digital mental health.

## Introduction

For mental health providers (MHPs), the burden of documentation presents a unique challenge that can impact both care quality and their well-being. While essential for continuity of care, the time required for meticulous charting often exceeds clinical hours and reduces direct patient engagement [1,2]. The pressure to complete timely documentation is a major driver of clinician burnout, as mental health professionals struggle to balance administrative demands with the emotional labor of therapeutic care [3-5]. Furthermore, inconsistent or poor-quality documentation increases cognitive load and threatens patient safety by obscuring critical information and hindering continuity of care [6,7].

To address documentation burden, solutions ranging from dictation and templates to medical scribes have emerged [8-10]. Human scribes, whether in person or virtual, reduce administrative time and increase direct patient interaction [11,12]. However, these methods can be costly, require extensive training, and face privacy and scalability issues due to high turnover [8,13]. Consequently, attention has shifted toward artificial intelligence (AI)–based digital scribe solutions. Early applications of AI in clinical documentation focused on natural language processing and machine learning for discrete tasks such as message triage. For instance, machine learning has been used to prioritize high-acuity patient portal messages, reducing clinician response delays and cognitive load [14,15]. In mental health, voice-to-text tools show promise for time savings but face hurdles regarding accuracy and workflow integration [16]. More recently, generative AI has been applied to session and discharge summarization, offering reduced clinician workload while necessitating careful oversight of note fidelity to combat issues such as AI hallucinations [17,18].

Building on these technologies, interest in virtual and AI scribes is growing to further reduce documentation burdens, and preliminary results in general health care settings are promising [8,9,18,19]. One study found that both clinicians and patients benefited from AI scribes, specifically highlighting clinicians’ enhanced ability to provide personalized care [18]. Similarly, a 2021 review identified key advantages of digital scribes, including easier charting, increased patient engagement, and improved care accessibility [8]. Research further indicates that these tools improve physician-patient interactions while producing high-quality notes requiring minimal editing [18]. While generative AI research in mental health is limited, emerging studies show similar promise for AI-assisted documentation. In psychiatry specifically, AI-generated notes required only 45% of the time of manual documentation while maintaining high clinician-rated accuracy and quality [20].

In April 2024, a HIPAA (Health Insurance Portability and Accountability Act)-compliant online therapy platform Talkspace launched Smart Notes, a generative AI tool designed specifically for MHPs. Here, we describe the development and deployment of the Smart Notes feature and evaluate MHP use, quality ratings, feedback and satisfaction, and productivity over its first year of implementation.

## Methods

### Overview

Talkspace matches individuals (referred to here as members) with an MHP licensed in the member’s state of residency. Talkspace employs both full-time and contractual (hourly) MHPs. Members, who may be able to access Talkspace via their insurance provider, employer, government program, or independently, are able to choose to connect with their MHP via asynchronous or live text, video, or audio sessions. Talkspace’s offerings include specialized individual therapy, couples therapy, and psychiatry. At the time of this analysis, Smart Notes had not yet been implemented for couples therapy, psychiatry, and certain live session plans. Therefore, the analysis includes only Smart Notes generated from eligible individual therapy sessions.

### Product Development and Deployment

The Smart Notes feature was developed using Azure OpenAI (Microsoft Corp) with a HIPAA-compliant infrastructure to securely process session data from live video transcripts, live audio transcripts, live messaging, and asynchronous messaging. Talkspace’s AI Governance Committee, a group composed of clinical, legal and regulatory compliance, product, engineering, and industry experts, reviewed the Smart Notes feature during the design phase to ensure usability, transparency, explainability, and privacy. Various prompts were iteratively tested and refined to optimize for note quality and clinical documentation standards. The initial prototype was tested on a dummy sample conversation prior to releasing the product to the initial pilot MHPs. We used text similarity scores that use the Dice-Sørenson coefficient [21] to determine the level of agreement between the generated draft note and the submitted note. We regarded text similarity scores under 90% as low. We used these scores to inform iterations of the model prompts until the average text similarity scores were consistently above 90%. To generate a Smart Note, session content first underwent a redaction process using a combination of commercially available (Amazon Web Services Glue’s entity detection; Amazon Web Services Inc) and open-source (python library “scrubadub”; Python Software Foundation) redaction software to ensure that all protected health information and personal identifying information were removed. The Talkspace app sent the deidentified contextual data, the deidentified treatment plan, and the prompt to the Azure application programming interface (API). The API then returned a draft of the clinical note, which the Talkspace app then presented to the therapist for review. The Azure API did not retain any data. Contextual data were saved on the Talkspace platform in an encrypted state.

Prior to use in a given therapy room, both the licensed therapist and individual member provided consent to the use of the Smart Notes feature to generate summarized documentation of therapy sessions. Following a session, MHPs had the option to request a Smart Note. MHPs were required to review the generated note and must make at least one edit to the generated note before submitting. To enforce this requirement, the system checked the generated note against the note the MHP was attempting to submit. If no edit was detected, the app presented a message to the MHP, prompting them to make at least one edit to ensure that MHPs were not passively accepting the AI-generated notes without any clinical input. After the edit was made, MHPs were able to submit the Smart Note as documentation of the session. Once the note was submitted, MHPs were given the option to rate and provide feedback on the note. Feedback was stored and evaluated by the team for further refinement of the feature.

Talkspace implemented a phased rollout and comprehensive training program to introduce Smart Notes to its MHP network. Following the feature’s announcement, MHPs attended live online training sessions featuring step-by-step demonstrations and interactive question-and-answer segments. To ensure continuous support, training materials were distributed via email and hosted in a central MHP help center, and monthly newsletters provided regular feature updates. Additionally, dedicated support teams were established to address ongoing inquiries. Early MHP feedback primarily focused on clinical and administrative guardrails, specifically regarding client-informed consent and protocols for transcription storage.

In an initial pilot period, a subset of Talkspace MHPs received early access to the feature. The chosen sample included full-time and hourly MHPs who were asked to provide critical, high-quality feedback necessary for the refinement and successful full-scale launch of the system. At launch, MHPs were able to use Smart Notes for asynchronous messaging sessions only, and live session availability was released 1 month later in May 2024. Smart Notes was launched to the entire MHP network for use in individual asynchronous client sessions as well as eligible individual live session plans in June 2024.

Finally, as part of an iterative product development and quality assurance process, the Smart Notes feature underwent a proof-of-concept validation led by 5 licensed clinicians (holders of degrees in licensed clinical social work and licensed mental health counseling) from the Talkspace Quality Management and Clinical Team. Clinicians evaluated the tool’s performance using a standardized clinical rubric. This rubric assessed 5 essential documentation pillars: documented diagnosis, high-risk member assessments, comprehensive mental health assessments, presenting problems, and defined treatment goals. This validation phase was intended to ensure consistency in meeting professional standards for clinical structure and content.

### Study Design

We conducted a retrospective observational mixed methods analysis using longitudinal and cross-sectional components to examine MHPs’ use of and experience with the Smart Notes tool. The study design integrated several quantitative measures collected consistently over time (Smart Notes use data, MHP ratings of note quality, generation error rates, text similarity scores, and MHPs’ net promoter score [NPS]); quantitative pre-post measures of MHPs’ productivity (eg, average weekly working hours); and qualitative open-ended feedback provided via quarterly MHP surveys.

### Data Collection

**Figure 1.**
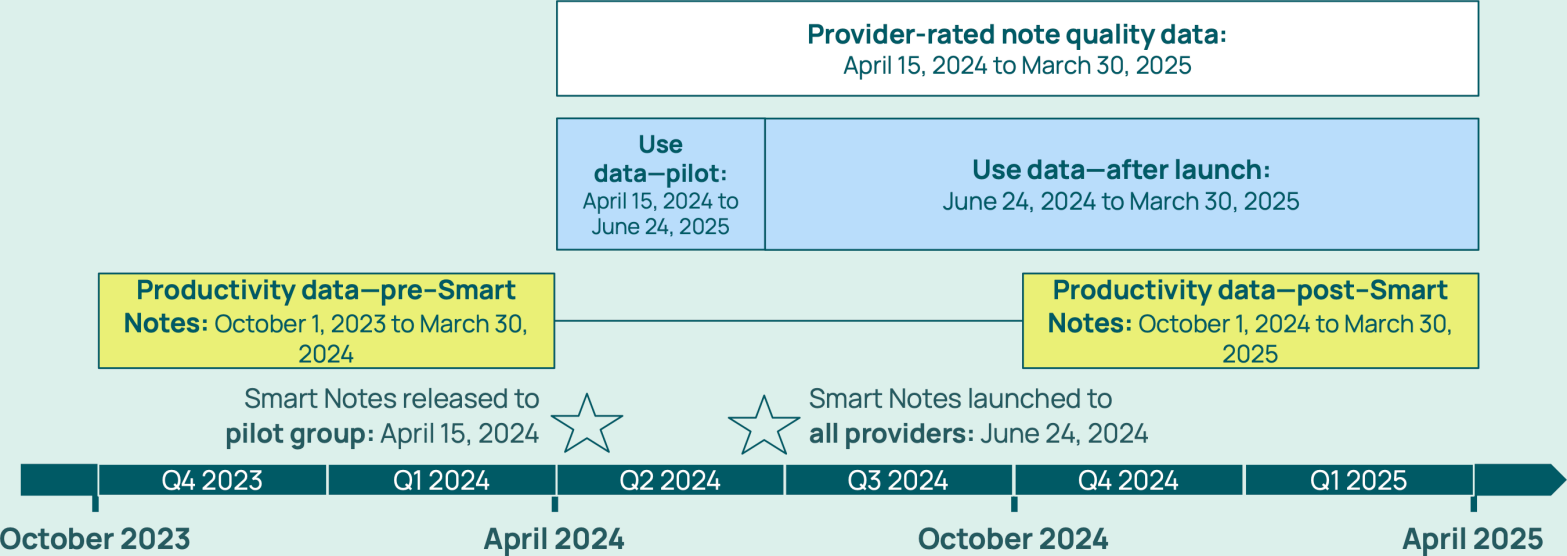
Data collection and analysis periods across the first, second, third, and fourth quarters of the year (Q1, Q2, Q3, and Q4, respectively).

Measures included MHPs’ Smart Notes use, note quality, generation error rates, text similarity scores, feedback and satisfaction, and productivity. A timeline of data collection is given in Figure 1.

#### Smart Notes Use

We examined Smart Notes use at the MHP level (ie, the number and percentage of MHPs who used Smart Notes each week, as compared with total MHPs) and at the note level (ie, the percentage of Smart Notes submitted each week, as compared with total progress notes) over time (Figure 1). We examined weekly use and uptake during the initial pilot period as well as after Smart Notes was launched to the entire MHP network. Notes were counted in the week the Smart Note was submitted, which could occur in a subsequent week after the session.

To examine the functionality over the same period, we also calculated the weekly percentage of generation errors and text similarity scores over the same period. Text similarity scores were calculated using the Dice-Sørenson coefficient [21], which gauges the similarity between two samples.

#### MHP-Rated Note Quality

Following each note submission, MHPs were prompted to provide optional feedback on the note with a “thumbs-up” or “thumbs down.” If a thumbs down was selected, indicating a negative rating, MHPs could then identify the reason for the negative rating from 3 options: “Too many edits needed,” “Summary is inaccurate,” and “Summary is non-formatted.” These data were collected for the year shown in Figure 1.

#### MHP Feedback and Satisfaction

Finally, we examined quantitative and qualitative MHP feedback from quarterly surveys that were sent to all Talkspace MHPs and could be submitted anonymously. Feedback included overall NPS (ie, not specific to Smart Notes) and open-ended, free-response feedback. For analyses of NPS and open-ended feedback data, all MHPs who submitted a survey response were included.

NPS is a metric used to gauge how likely or unlikely respondents are to recommend a given platform, product, or company based on a single question regarding respondent satisfaction. NPS was calculated using MHPs’ 1 to 10 ratings on the question “How likely are you to recommend Talkspace to a colleague?” by calculating the percentage of “promoters” (scores of 9 or 10; those who are satisfied and will recommend the business) minus the percentage of “detractors” (scores of 0 through 6; those who are unsatisfied and might actively discourage other users). We examined whether MHPs’ NPS on the Talkspace platform as a whole differed between MHPs who did and did not use Smart Notes.

MHPs’ free-response feedback about Talkspace was collected quarterly alongside NPS. We examined Smart Notes–related themes in MHPs’ responses submitted during the quarters after the feature had been fully launched to all MHPs, reporting the number and percent of MHPs endorsing each theme.

#### MHP Productivity

Finally, to assess MHP productivity, we calculated MHPs’ total weekly working hours, completed sessions, and weekly caseload. Total working hours were defined as the sum of hours spent on video calls, hours spent on SMS text messaging (calculated from the number of messages at 40 words per minute), hours of voice or video messages sent, and (for full-time MHPs) hours of vacation time. Completed sessions were defined as the sum of the live video sessions and asynchronous messaging sessions. Weekly caseload was defined as the count of clients that a MHP interacted with in a given week. We then compared MHPs’ productivity after the introduction of Smart Notes (post–Smart Notes) vs before Smart Notes existed (pre–Smart Notes; Figure 1). We defined a 6-month post–Smart Notes period after the feature had been fully rolled out to ensure MHP uptake had steadied. We used the same 6-month period 1 year prior as the pre–Smart Notes period for comparison to minimize potential effects of time of year. To examine this pattern over time, independent of the introduction of Smart Notes, we replicated these analyses for the previous year (October 2022-March 2023 compared with October 2023-March 2024) and for the current period with MHPs who had been active since 2022. These results are presented in Multimedia Appendix 1.

### Data Analyses

**Figure 2.**
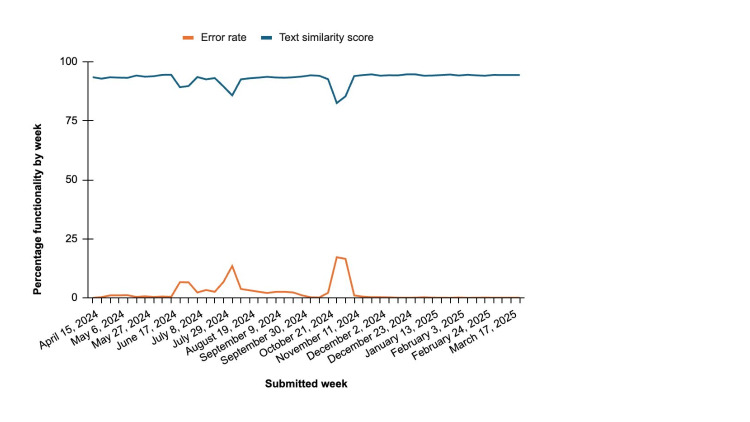
Smart Note functionality by week: generation error rate and text similarity scores.

For analysis of MHP use, error rates, similarity scores, note quality, and productivity, we included data from Talkspace MHPs who met the following criteria: (1) MHPs were consistently employed at Talkspace for the study period (6 quarters around deployment of Smart Notes shown in Figure 1) and (2) MHPs conducted at least 1 asynchronous or live therapy session during each of two 6-month periods before and after Smart Notes (Figure 1). For productivity comparison analyses, we also excluded MHPs who switched employment status within Talkspace (eg, from full time to contractual) at any time during the study period. Where indicated, MHP types (full time and contractual) were analyzed and reported separately because these MHPs differed in weekly workload requirements.

Smart Notes use, error rates, text similarity scores, and MHP-rated note quality are examined via descriptive statistics (counts and percents). MHP productivity was examined across total working hours, completed sessions, and client caseload using within-subjects 2-tailed *t* tests. MHP feedback and satisfaction data were presented via between-subjects 2-tailed *t* tests (NPS) and manual, data-driven thematic analysis (open-ended survey feedback) [22,23]. Open-ended survey responses were first marked for references to various product features, including Smart Notes, and positive or negative feedback about each feature mentioned. Next, all responses that mentioned Smart Notes were read and initial notes (ie, codes) were generated for each. These codes were then categorized into 6 overarching themes, with a final read to verify that the assigned themes fit each comment. Each open-ended response could receive one or more theme codes.

### Ethical Considerations

All data were collected as part of ongoing business operations between October 2023 and March 2025 and were deidentified prior to analysis. The use of participant data for this research was reviewed and deemed exempt by the Advarra Institutional Review Board under 45 Code of Federal Regulations §46.104(d)(4) (case Pro00084177/TS-001, dated June 2025) [24]. Members and MHPs gave consent for the use of their deidentified data for research and product development purposes by accepting the platform’s privacy policy at account creation. Model development was based on deidentified therapy transcripts, which remained in an encrypted Talkspace database and were not accessed by the researchers at any time. No compensation was provided to the participants.

## Results

### Smart Notes Use

#### Overview

Across nearly 1 year (Figure 1), 162 unique full-time MHPs submitted 88,591 Smart Notes, and 1366 unique contractual MHPs submitted 198,320 Smart Notes. The total number of Smart Notes was higher than traditional progress notes submitted over the same period—full-time MHPs submitted 51,345 traditional progress notes, and contractual MHPs submitted 186,610. Over the same period, the average generation error rate per week was 2% (SD 4%; range 0%-17%). However, during most weeks, the error generation rate was less than 0.2%. Following a similar pattern, the text similarity scores averaged 93% (SD 3%; range 83%-95%) per week, with slight dips during weeks with higher generation errors (Figure 2). These dips were due to technical service outages on the part of either Talkspace or its vendors.

#### Pilot

**Figure 3.**
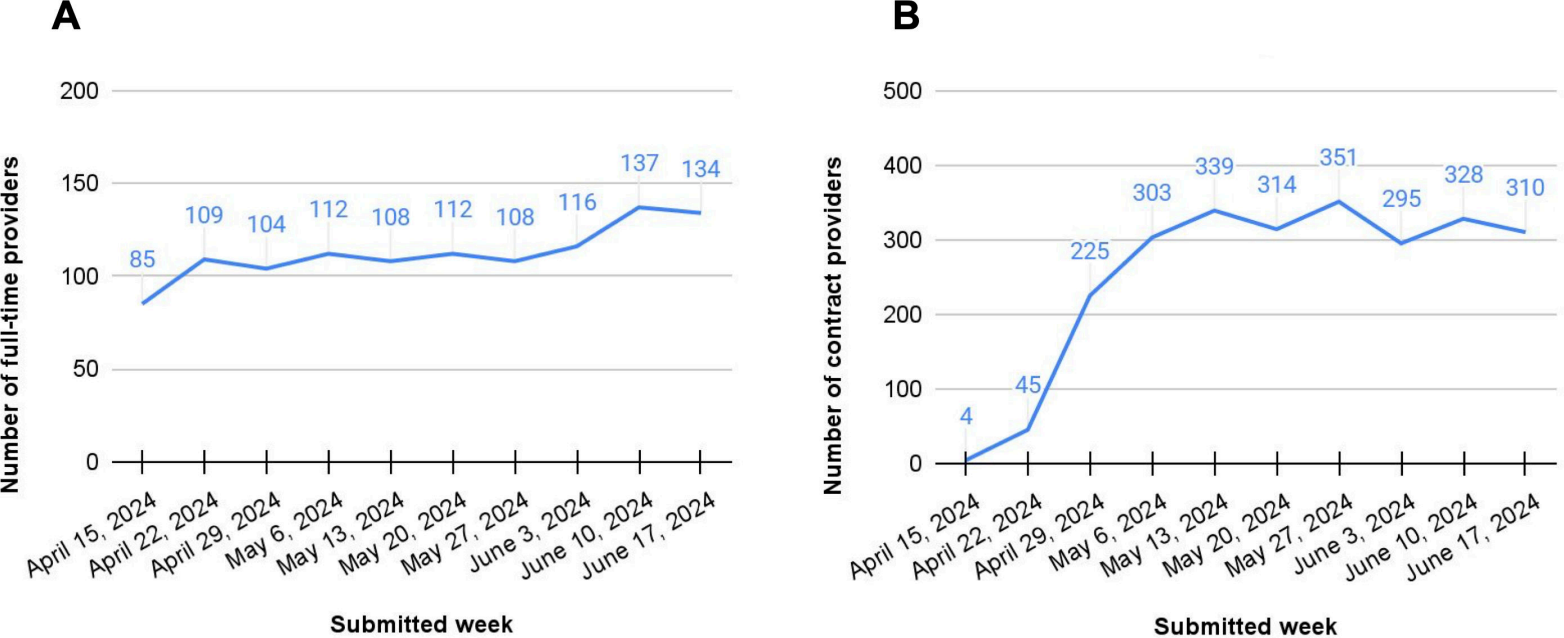
(A) Full-time and (B) contractual mental health providers using Smart Notes during the pilot period.

Among the subset of Talkspace MHPs who received early access to pilot the Smart Notes feature, uptake grew quickly to more than 100 full-time MHPs (Figure 3A) and 300 contractual MHPs (Figure 3B) using the feature each week during the pilot period.

**Figure 4.**
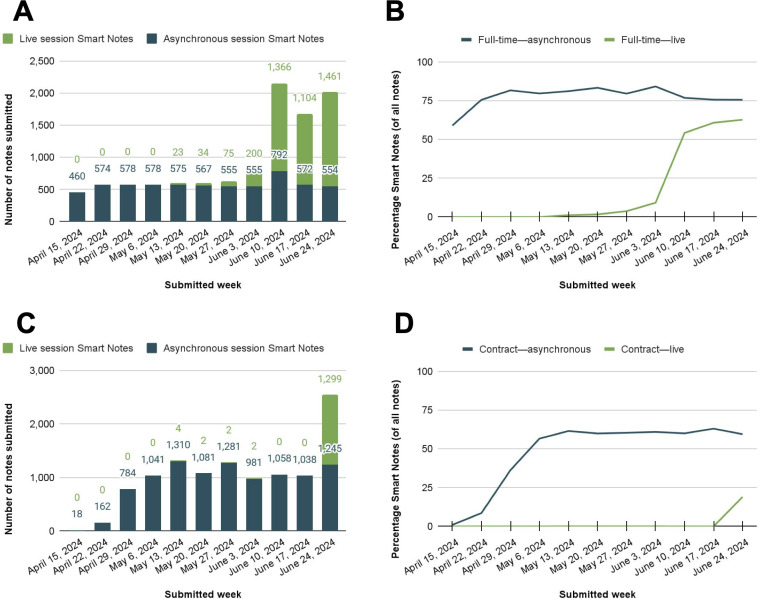
Smart Notes submitted by mental health providers (MHPs) during the pilot period. (A) Count of full-time MHPs' Smart Notes by week; (B) percentage of Smart Notes submitted by full-time MHPs each week; (C) count of contractual MHPs' Smart Notes by week; and (D) percentage of Smart Notes submitted by contractual MHPs each week.

Among full-time MHPs, Smart Notes use increased rapidly after the feature became available and held steady at an average of 80% of MHPs’ notes for asynchronous sessions (Figure 4A, B). Full-time MHPs also used Smart Notes for live sessions as soon as the feature became available, growing quickly to 60.8% (1104/1815) of MHPs’ live progress notes in the week before full launch. Among contractual MHPs, Smart Notes use grew to 56.7% (1041/1837) of MHPs’ asynchronous progress notes by the fourth week, then held steady thereafter (Figure 4C, D). Contractual MHPs did not receive access to Smart Notes for live sessions during the pilot period.

#### After Launch

**Figure 5.**
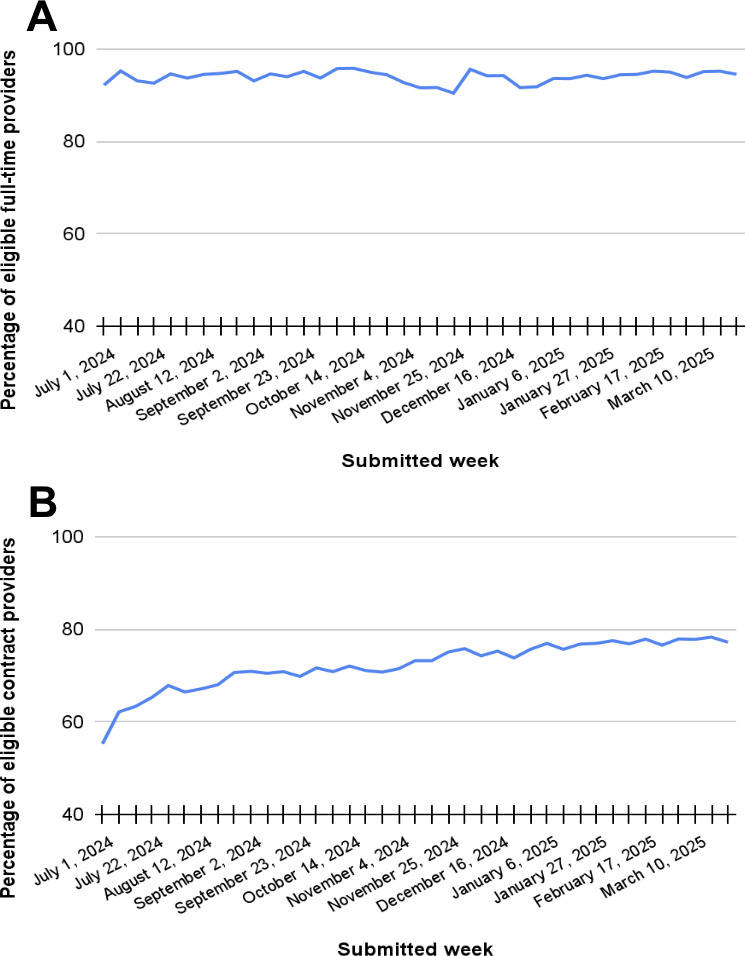
(A) Fut to ull-time and (B) contractual mental health providers using Smart Notes after launch.

**Figure 6.**
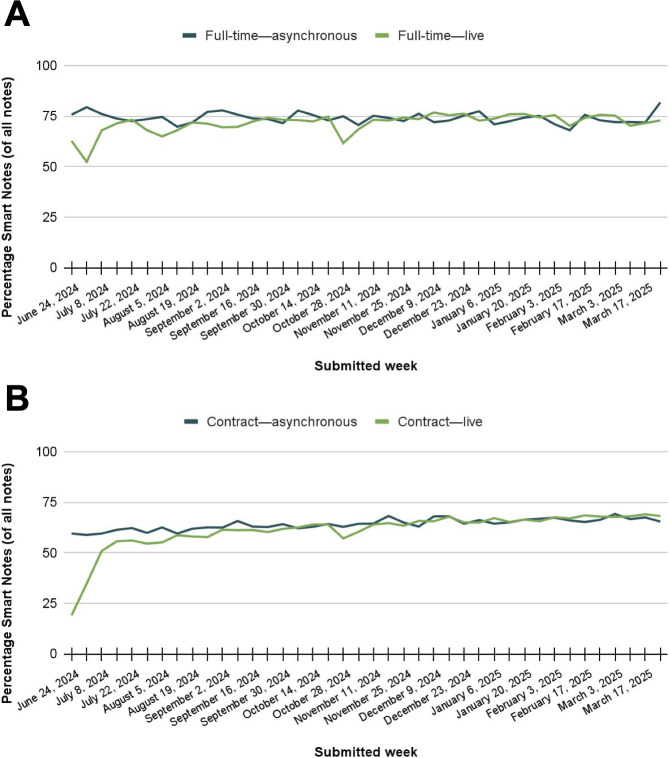
Smart Notes submitted by (A) full-time and (B) contractual mental health providers during the weeks after launch.

Following the pilot period, Smart Notes was made available to all MHPs on the Talkspace platform for individual asynchronous therapy sessions and eligible live therapy sessions. At launch, percent use was high among both full-time MHPs (Figure 5A) and contractual MHPs (Figure 5B). Among full-time MHPs, 96% (142/148) used the Smart Notes feature during the first week of its full launch across the platform. Use remained high over time, with nearly all MHPs using Smart Notes on a weekly basis (mean 94%, SD 1%; Figure 5A). Full-time MHPs averaged 2000 (SD 283.7) Smart Notes per week as a group or averaged 72% (SD 3%) of all progress notes they submitted (Figure 6A). Additional data broken out by asynchronous and live sessions are given in Table 1.

Among contractual MHPs, 51% (607/1181) used the Smart Notes feature during the first week of its full launch across the platform. Over time, uptake increased to an average of 72% (SD 6%) of all MHPs (Figure 5B). Contractual MHPs averaged 4739 (SD 784.7) Smart Notes per week as a group or 62% (SD 8%) of all contractual MHPs’ progress notes (Figure 6B). Table 1 shows data broken out by asynchronous and live sessions.

**Table 1.** Weekly average Smart Notes submitted by mental health providers (MHPs) after launch.

	Full-time MHPs				Contractual MHPs			
	Mean (SD)		Range		Mean (SD)		Range	
All sessions								
Smart Notes, n	1999.6 (283.7)		1034.0-2425.0		4738.9 (784.7)		2328.0-5755.0	
Smart Notes (%)	72 (3)		59-76		62 (8)		29-69	
Asynchronous sessions								
Smart Notes, n	453.7 (84.7)		268.0-666.0		919.2 (145.6)		704.0-1307.0	
Smart Notes (%)	74 (3)		68-82		64 (3)		59-69	
Live sessions								
Smart Notes, n	1545.9 (242.7)		766.0-1897.0		3819.7 (805.8)		1299.0-4789.0	
Smart Notes (%)	72 (5)		52-77		61 (9)		19-69	

### MHP-Rated Note Quality

Smart Note quality was monitored on an ongoing basis. Out of 466,139 total Smart Notes, MHPs gave optional feedback ratings on 30,519 (6.5%). Of these, Smart Notes were nearly all rated “thumbs-up” (positive): 97.7% (9980/10,219) of full-time MHPs’ ratings and 98.4% (19,977/20,300) of contractual MHPs’ ratings were positive. Most negative ratings (n=562, 1.8%) were related to summary inaccuracy (150/323, 46.4% ratings from contractual MHPs and 125/239, 52.3% from full-time MHPs) or too many edits needed (135/323, 41.8% ratings from contractual MHPs and 97/239, 40.6% from full-time MHPs).

### MHP Feedback and Satisfaction

Contractual MHPs who used Smart Notes (167/421, 39.7%; mean 7.25, SD 2.68) gave NPS ratings of Talkspace that were no different from those who did not use Smart Notes (254/421, 60.3%; mean 6.96, SD 3.11; *t*_389.92_=1.022; *P*=.31). Similarly, full-time MHPs who used Smart Notes (39/73, 53.4%; mean 8.46, SD 2.21) also gave NPS ratings of Talkspace that were no different from those who did not use Smart Notes (34/73, 46.6%; mean 8.88, SD 1.39; *t*_64.854_=0.987; *P*=.33).

Quarterly surveys, which included opportunities to give anonymous open-ended feedback about Talkspace, included many comments about Smart Notes. A total of 128 full-time MHPs and 685 contractual MHPs gave open-ended feedback in Q3 2024, 197 and 668 in Q4 2024, and 156 and 632 in Q1 2025. Among those respondents, the Smart Notes feature was mentioned by 3 (2.3%) full-time MHPs and 30 (4.4%) contractual MHPs in Q3 2024, 9 (4.6%) and 28 (4.2%) in Q4 2024, and 23 (14.8%) and 28 (4.4%) in Q1 2025.

Across quarters, MHPs’ Smart Notes feedback was overall positive (120/126, 95.2% positive vs 6/126, 4.8% negative comments; Table 2), including general praise (eg, “I love the Smart Notes!”), specific praise (eg, saves time, makes work easier), and requests for the feature’s expansion across the platform (eg, Smart Notes for couples sessions or sessions conducted in Spanish). Few comments mentioned issues with or suggested improvements to Smart Notes (eg, concerns about privacy and comparison to competitors’ features).

**Table 2.** Summary of Smart Notes feedback in open-ended mental health provider (MHP) responses^a^.

Theme	Full-time MHPs (n=33), n (%)	Contractual MHPs (n=82), n (%)
General praise for Smart Notes	6 (18.2)	43 (52.4)
Smart Notes saves MHPs time	4 (12.1)	4 (4.9)
Smart Notes makes MHPs’ work easier	3 (9.1)	3 (3.7)
Request for Smart Notes to expand (eg, for use in couples’ sessions)	26 (78.8)	31 (37.8)
Artificial intelligence privacy concerns	1 (3.0)	2 (2.4)
Issues or improvements	0 (0.0)	3 (3.7)

a Percentages do not add to 100 because MHPs could have mentioned more than one of these themes in their responses.

### MHP Productivity

Table 3 presents data on full-time (n=140) and contractual (n=1698) MHPs in the pre–Smart Notes and post–Smart Notes periods. For additional context, data from a second pre–Smart Notes period replicating the main results are included in Multimedia Appendix 1.

**Table 3.** Weekly average working hours, completed sessions, and clients pre–Smart Notes and post–Smart Notes.

	Pre–Smart Notes, mean (SD)		Post–Smart Notes, mean (SD)	
Full-time mental health providers				
Hours	18.2 (8.15)		18.6 (8.93)	
Sessions	15.5 (7.64)		16.5 (8.41)	
Clients	18.0 (6.10)		19.4 (6.61)	
Contractual mental health providers				
Hours	4.11 (5.80)		4.11 (6.08)	
Sessions	3.91 (5.55)		4.06 (6.10)	
Clients	5.48 (5.86)		5.91 (6.70)	

Total working hours—neither full-time MHPs (*t*_139_=1.263; *P*=.21) nor contractual MHPs (*t*_1697_=0.175; *P*=.86) demonstrated a change in their total working hours between the 2 periods.

Completed sessions—both full-time MHPs (*t*_139_=2.592; *P*=.01) and contractual MHPs (*t*_1697_=2.053; *P*=.04) demonstrated a significant increase in weekly sessions completed from pre–Smart Notes to post–Smart Notes.

Clients—both full-time MHPs (*t*_138_=3.496; *P*<.001) and contractual MHPs (*t*_1440_=2.735; *P*=.006) demonstrated a significant increase in weekly caseload from pre–Smart Notes to post–Smart Notes.

## Discussion

### Principal Findings

This study aimed to describe the development and application of Talkspace’s Smart Notes, a generative AI tool designed to assist with documentation tasks for MHPs. Our findings provide initial evidence that AI-powered documentation tools are a feasible and well-received solution for supporting mental health clinicians, in line with existing work in other medical specialties, including psychiatry [18-20]. However, to the best of our knowledge, this is the first study to specifically investigate the utility of an AI scribe service within a large-scale digital mental health care context. The results shed light on how such technology may be used in administrative workflows and its association with MHP productivity and satisfaction, highlighting a potential avenue to ultimately support access to mental health services.

### Interpretation of Key Findings

Three key findings from this preliminary investigation are particularly significant. First, the rapid and sustained uptake of Smart Notes suggests that it aligns with MHP interest and demand. The consistent use, with nearly all full-time MHPs and most contractual MHPs using the feature weekly for eligible sessions, indicates that the tool was compatible with MHPs’ existing workflows rather than disrupting them [16,18]. Notably, full-time MHPs adopted the feature at higher rates than hourly contractual MHPs, which may be due to their more consistent, full-time caseloads.

Second, the high-quality ratings of the Smart Notes were a critical factor in their acceptability. Nearly all note quality ratings were positive (9980/10,219, 97.7% among full-time MHPs and 19,977/20,300, 98.4% among contractual MHPs), suggesting that MHPs perceived the tool as generating accurate and useful session summaries, a prerequisite for widespread adoption in a clinical setting. The low rate of negative feedback related to accuracy and formatting indicates that the tool performed consistently with clinical standards. The required human review before submission ensured that any Smart Note not meeting clinical standards was edited or corrected by the MHP, maintaining clinical integrity and MHP accountability. High text similarity scores between the generated Smart Note and the MHP-reviewed submitted note indicate that MHPs did not need to make many edits. Ethically, the reliance on MHP review underscores the importance of the “human-in-the-loop” model. Although very few MHPs reported inaccuracies and the submitted notes were consistent with Smart Notes, the requirement for MHP sign-off aligns with recommendations for responsible AI deployment [17,18], ensuring that clinical oversight remains central to documentation.

In addition, the overall positive qualitative feedback on Smart Notes contextualizes the high use rates and further reinforces the tool’s value. MHPs praised Smart Notes for saving them time and making their work easier, 2 core pain points of documentation burden that the tool was initially created to address. The strong positive reception of this specific feature suggests that targeted technological interventions can garner favorable MHP perceptions of their workload within a larger digital health care ecosystem.

Finally, our data also show that Smart Notes implementation coincided with trends in MHP productivity. Although small, the observed increase in completed sessions following the feature’s introduction, without a corresponding increase in MHPs’ working hours, aligns with prior research suggesting that a reduced documentation burden can improve productivity by freeing up MHPs’ time for client-facing activities [8,18,19], ultimately increasing access to care. This finding is especially relevant for a field struggling with MHP capacity and retention.

### Implications and Limitations

Although this study offers valuable insights, it is important to consider its limitations. The primary limitation is the observational design, which prevents us from establishing a causal link between the use of Smart Notes and the observed increase in MHP session completion. It is possible (and indeed, our supplemental analysis supports) that confounding factors, such as natural caseload growth, other changes to the Talkspace platform, or various external factors such as seasonality may have driven these productivity metrics independent of the Smart Notes tool. Future research might survey MHPs directly for reflections on how Smart Notes impacted their workflows and perceived documentation burden and productivity. Additional research is also needed to isolate the effects of Smart Notes use on MHPs’ productivity over time.

Additionally, although we were able to assess the change in MHPs’ client-facing working hours, administrative and documentation time was not measurable on the platform, limiting our ability to detect a difference in time spent on non–client-facing tasks. However, measurement of administrative time is subject to substantial variability (eg, MHPs could leave the platform open while not actively working on a task, and multiple types of tasks are included in administrative time). Focusing on shifts in the number of client-facing hours provides an early metric for tracking potential associations between Smart Notes and productivity.

Finally, the high satisfaction ratings (120/126, 95.2%) and qualitative feedback may be subject to nonresponse or extremity bias [25], particularly given the small percentage of notes (30,519/466,139, 6.5%) with submitted formal quality ratings. However, the data did not exhibit the bimodal “U-shaped” distribution typical of extremity bias [26], and negative feedback remained minimal despite multiple anonymous feedback channels. The validity of these positive ratings is further supported by high uptake and sustained longitudinal use, suggesting that MHP behavior aligns with the reported satisfaction levels. Positive feedback results also align with existing proof-of-concept research with psychiatrists [20] and psychiatrists’ general views of AI documentation in mental health [27].

Our findings have important practical implications for both MHPs and mental health care organizations. This study provides preliminary evidence that AI tools can be feasible and acceptable to MHPs and garner favorable perceptions related to documentation burden, potentially supporting their capacity to deliver care. For organizations, investing in these technological solutions may be relevant for addressing MHP burnout, satisfaction, and retention. As AI tools become more integrated into mental health care, continued research is essential to understand their immediate and long-term impact on MHP well-being, patient outcomes, and the evolving nature of documentation practices.

### Conclusions

This retrospective observational study suggests that AI-powered documentation tools such as Smart Notes can be successfully integrated into digital mental health workflows to support MHPs. High adoption rates, coupled with favorable MHP feedback, underscore the potential utility of these tools in assisting with administrative demands while maintaining clinical standards. Overall, this study contributes preliminary data regarding the deployment of generative AI in a behavioral health context, highlighting a promising avenue for future research into scalable solutions to address documentation burden.

## Supplementary material

10.2196/84628 Multimedia Appendix 1 Mental health provider productivity from 2022 onward.

## Abbreviations

AI: artificial intelligence
API: application programming interface
HIPAA: Health Insurance Portability and Accountability Act
MHP: mental health provider
NPS: net promoter score

## References

[1] Griffin NE, Nour SI, Udayappan KM, Bonnes SL, Sawatsky AP, Croghan IT (2025). Physician documentation workflow preferences and perceptions. Discov Health Syst.

[2] Tran B, Lenhart A, Ross R, Dorr DA (2019). Burnout and EHR use among academic primary care physicians with varied clinical workloads. AMIA Jt Summits Transl Sci Proc.

[3] Tajirian T, Stergiopoulos V, Strudwick G (2020). The influence of electronic health record use on physician burnout: cross-sectional survey. J Med Internet Res.

[4] Isenberg BM, Chu W, Boyd MR (2025). A qualitative study of school mental health providers’ experiences with chart notes: perceived utility, burden, and areas for growth. Evid Based Pract Child Adolesc Ment Health.

[5] Murthy VH (2022). Confronting health worker burnout and well-being. N Engl J Med.

[6] Vetter CD, Kim JH (2023). Impact of implementing structured note templates on data capture for hernia surgery. Health Inf Manag.

[7] Feldman J, Goodman A, Hochman K (2023). Novel note templates to enhance signal and reduce noise in medical documentation: prospective improvement study. JMIR Form Res.

[8] Ghatnekar S, Faletsky A, Nambudiri VE (2021). Digital scribe utility and barriers to implementation in clinical practice: a scoping review. Health Technol (Berl).

[9] Hudelson C, Gunderson MA, Pestka D (2024). Selection and implementation of virtual scribe solutions to reduce documentation burden: a mixed methods pilot. AMIA Jt Summits Transl Sci Proc.

[10] Calder MB, Hanson M, Jost M, Kelley KD (2024). Time and note characteristic effects of an electronic health record template for internal medicine resident notes. J Grad Med Educ.

[11] Hartman-Hall H, Kanwal A, Jory L (2023). Scribes with PGY-1 residents on inpatient medicine teams: effect on time spent in meaningful work. J Community Hosp Intern Med Perspect.

[12] Duggan MJ, Gervase J, Schoenbaum A (2025). Clinician experiences with ambient scribe technology to assist with documentation burden and efficiency. JAMA Netw Open.

[13] Agarwal P, Lall R, Girdhari R (2024). Artificial intelligence scribes in primary care. CMAJ.

[14] Cronin RM, Fabbri D, Denny JC, Rosenbloom ST, Jackson GP (2017). A comparison of rule-based and machine learning approaches for classifying patient portal messages. Int J Med Inform.

[15] Yang J, So J, Zhang H (2024). Development and evaluation of an artificial intelligence-based workflow for the prioritization of patient portal messages. JAMIA Open.

[16] Fernandes J, Brunton I, Strudwick G, Banik S, Strauss J (2018). Physician experience with speech recognition software in psychiatry: usage and perspective. BMC Res Notes.

[17] Schwieger A, Angst K, de Bardeci M (2024). Large language models can support generation of standardized discharge summaries - a retrospective study utilizing ChatGPT-4 and electronic health records. Int J Med Inform.

[18] Tierney AA, Gayre G, Hoberman B (2024). Ambient artificial intelligence scribes to alleviate the burden of clinical documentation. NEJM Catal Innov Care Deliv.

[19] Ma SP, Liang AS, Shah SJ (2025). Ambient artificial intelligence scribes: utilization and impact on documentation time. J Am Med Inform Assoc.

[20] Stanton N, Aziz A, Jakhra S (2025). Evaluating artificial intelligence ambient voice technology as a documentation assistant in psychiatry: proof-of-concept study. BJPsych Bull.

[21] Warrens MJ (2008). On similarity coefficients for 2x2 tables and correction for chance. Psychometrika.

[22] Braun V, Clarke V (2006). Using thematic analysis in psychology. Qual Res Psychol.

[23] Ferrario B, Stantcheva S (2022). Eliciting people’s first-order concerns: text analysis of open-ended survey questions. AEA Pap Proc.

[24] (2024). Exempt Research. Code of Federal Regulations, title 45 (2024): sec. 46.104. Code of Federal Regulations.

[25] Lavrakas PJ, Lavrakas PJ (2008). Encyclopedia of Survey Research Methods.

[26] Ullah R, Amblee N, Kim W, Lee H (2016). From valence to emotions: exploring the distribution of emotions in online product reviews. Decis Support Syst.

[27] Blease C, Worthen A, Torous J (2024). Psychiatrists’ experiences and opinions of generative artificial intelligence in mental healthcare: an online mixed methods survey. Psychiatry Res.

[28] Suchikova Y, Tsybuliak N, Teixeira da Silva JA, Nazarovets S (2026). GAIDeT (Generative AI Delegation Taxonomy): a taxonomy for humans to delegate tasks to generative artificial intelligence in scientific research and publishing. Account Res.

