# Supplementary material for: AI-Powered Documentation for Mental Health Providers: Retrospective Observational Mixed Methods Study
Source: JMIR Form Res. 2026 Mar 12;10:e84628. doi: 10.2196/84628 (PMC12981374; doi:10.2196/84628)
Supplement: Multimedia Appendix 1 [file formative-v10-e84628-s001.docx]

**Supplementary File 1**: MHP Productivity from 2022 Onward

**Table S1**. Replication of main results with MHPs who were active between October 2022 and March 2025

|  | **Pre-Smart Notes**  **Oct 2023-March 2024** | | **Post-Smart Notes**  **Oct 2024-March 2025** | |  |
| --- | --- | --- | --- | --- | --- |
|  | ***M*** | ***SD*** | ***M*** | ***SD*** | ***t*** |
| *Full-Time MHPs* | | | | |  |
| **Hours** | 16.98 | 7.01 | 17.43 | 7.20 | *t* = 1.012,  *df* = 62,  *p* = 0.316 |
| **Sessions** | 13.74 | 5.70 | 14.86 | 5.91 | *t* = 2.794,  *df* = 62,  *p* = .010 |
| **Clients** | 16.41 | 6.69 | 18.22 | 7.21 | *t* = 4.015,  *df* = 62,  *p* < .001 |
| *Contract MHPs* | | | | |  |
| **Hours** | 4.12 | 5.22 | 4.13 | 5.59 | *t* = 0.021,  *df* = 878,  *p* = .983 |
| **Sessions** | 3.72 | 4.52 | 3.94 | 5.26 | *t* = 2.454,  *df* = 878,  *p* = .014 |
| **Clients** | 5.22 | 5.31 | 5.67 | 6.27 | *t* = 2.687,  *df* = 753,  *p* = 0.007 |

**Table S2.** Replication of main results from October 2022 to March 2024 (before Smart Notes were introduced) in MHPs who were active between October 2022 and March 2025

|  | **Baseline**  **Pre-Smart Notes**  **Oct 2022-March 2023** | | **Pre-Smart Notes**  **Oct 2023-March 2024** | |  |
| --- | --- | --- | --- | --- | --- |
|  | ***M*** | ***SD*** | ***M*** | ***SD*** | ***t*** |
| *Full-Time MHPs* | | | | |  |
| **Hours** | 15.48 | 5.06 | 16.97 | 7.06 | *t* = 2.09,  *df* = 61,  *p* = 0.040 |
| **Sessions** | 10.62 | 3.68 | 13.76 | 5.74 | *t* = 4.83,  *df* = 61,  *p* < .001 |
| **Clients** | 13.65 | 4.38 | 15.45 | 6.73 | *t* = 3.57,  *df* = 61,  *p* < .001 |
| *Contract MHPs* | | | | |  |
| **Hours** | 4.21 | 4.77 | 4.18 | 5.26 | *t* = 0.174,  *df* = 858,  *p* = .862 |
| **Sessions** | 3.54 | 4.14 | 3.77 | 4.55 | *t* = 1.990,  *df* = 858,  *p* = .047 |
| **Clients** | 5.24 | 5.06 | 5.26 | 5.35 | *t* = 0.177,  *df* = 757,  *p* = 0.859 |
